# Bark Transpiration Rates Can Reach Needle Transpiration Rates Under Dry Conditions in a Semi-arid Forest

**DOI:** 10.3389/fpls.2021.790684

**Published:** 2021-12-20

**Authors:** Anna Lintunen, Yakir Preisler, Itay Oz, Dan Yakir, Timo Vesala, Teemu Hölttä

**Affiliations:** ^1^Institute for Atmospheric and Earth System Research/Physics, University of Helsinki, Helsinki, Finland; ^2^Institute for Atmospheric and Earth System Research/Forest Sciences, University of Helsinki, Helsinki, Finland; ^3^Department of Earth and Planetary Sciences, Weizmann Institute of Science, Rehovot, Israel; ^4^Laboratory of Ecosystem-Atmospheric Interactions of Forest - Mire Complexes, Yugra State University, Khanty-Mansiysk, Russia

**Keywords:** drought, embolism, irrigation experiment, lenticel, *Pinus halepensis*, stem respiration

## Abstract

Drought can cause tree mortality through hydraulic failure and carbon starvation. To prevent excess water loss, plants typically close their stomata before massive embolism formation occurs. However, unregulated water loss through leaf cuticles and bark continues after stomatal closure. Here, we studied the diurnal and seasonal dynamics of bark transpiration and how it is affected by tree water availability. We measured continuously for six months water loss and CO_2_ efflux from branch segments and needle-bearing shoots in *Pinus halepensis* growing in a control and an irrigation plot in a semi-arid forest in Israel. Our aim was to find out how much passive bark transpiration is affected by tree water status in comparison with shoot transpiration and bark CO_2_ emission that involve active plant processes, and what is the role of bark transpiration in total tree water use during dry summer conditions. Maximum daily water loss rate per bark area was 0.03–0.14 mmol m^−2^ s^−1^, which was typically ~76% of the shoot transpiration rate (on leaf area basis) but could even surpass the shoot transpiration rate during the highest evaporative demand in the control plot. Irrigation did not affect bark transpiration rate. Bark transpiration was estimated to account for 64–78% of total water loss in drought-stressed trees, but only for 6–11% of the irrigated trees, due to differences in stomatal control between the treatments. Water uptake through bark was observed during most nights, but it was not high enough to replenish the lost water during the day. Unlike bark transpiration, branch CO_2_ efflux decreased during drought due to decreased metabolic activity. Our results demonstrate that although bark transpiration represents a small fraction of the total water loss through transpiration from foliage in non-stressed trees, it may have a large impact during drought.

## Introduction

Changes in the frequency, duration and/or severity of drought and heat stress associated with climate change (IPCC, 2021) could fundamentally alter the composition and structure of forests in many regions (Allen et al., 2010; Choat et al., 2012). Potential reductions in growth and increases in tree mortality associated with climate-induced physiological stress and interactions with other climate-mediated processes, such as insect outbreaks and pathogens, are of particular concern (Allen et al., 2010; Brodribb et al. 2020; Lloyd and Bunn, 2007; Scholze, et al., 2006). Extreme drought stresses and kills trees through excessive embolism formation, and prolonged water stress may lead to carbon starvation due to closed stomata and metabolic limitations (McDowell et al., 2013; Meir, et al. 2015; Mencuccini, et al. 2015; Salmon et al., 2015). Maintaining a functional xylem network is so critical to survival that plants typically prioritise preventing water loss over carbon gain through stomatal closure before massive embolism formation occurs (see Delzon and Cochard, 2014). This so-called hydraulic safety margin, i.e., the difference between the level of water stress experienced by a species in the field and the level of water stress leading to hydraulic failure, is generally higher in gymnosperms than in angiosperms, but most plant species live on the verge of hydraulic failure with surprisingly small hydraulic safety margins (Choat et al., 2012). In the case of extreme heat and drought, the hydraulic safety margin may be even further reduced due to unregulated water loss through leaf cuticles and stem bark after stomatal closure (Cochard, 2019; Cochard, et al. 2021).

When the stomata are fully closed, water can still exit the leaves to the atmosphere through the leaf cuticle (Machado et al., 2020). The cuticle is a non-cellular and hydrophobic film containing cuticular waxes. Cuticular transpiration can be measured from isolated cuticles, leaf sides without stomata or by measuring the minimum leaf conductance in conditions with maximum stomatal closure. In the latter case, it is more accurate to talk about residual or minimum transpiration after maximum stomatal closure, as the stomata may leak even when they are seemingly closed (Burghardt and Riederer, 2003; Dayer et al., 2020; Machado et al., 2020). Minimum leaf transpiration is increasingly recognised to play an important role during heat waves (Kala et al., 2016) and in models of plant drought response (Blackman, et al., 2016; Cochard et al., 2021; Martin-StPaul, et al., 2017). Meta-analyses with various plant life forms show that the minimum leaf conductance after presumed stomatal closure does not seem to differ significantly between deciduous trees and evergreen conifers (Duursma, et al., 2019; Schuster, et al., 2017). Unlike stomatal transpiration, cuticular transpiration cannot be actively regulated by plants during drought, but cuticular permeability to water may decline with decreasing leaf water content due to shrinking of the cuticle (Anfodillo, et al., 2002; see Duursma et al., 2019; van Gardingen and Grace, 1992).

After leaf stomata are closed, water can also be lost from the tree to the ambient air through the bark. In plant parts that experience secondary growth, such as tree branches and stems, the protective barrier around their aerial tissues to avoid uncontrolled water loss is provided by the outer bark, also called the periderm. The periderm consists of cork, the cork cambium and the phelloderm. The cork is practically impermeable to gases and water, but it is pierced by lenticels that provide channels for water and gas exchange with the ambient air to allow metabolic processes in the living cells of the stem (Lendzian, 2006, Figs. 134 & 135 in Angyalossy et al., 2016). Literature on lenticels in tree branches and stems is scarce, but the current knowledge reports, for example that ~1–4% of the bark surface in *Picea abies* [(L.) H. Karst] is covered by lenticels (Rosner and Kartusch, 2003), and the permeability of water and oxygen has been shown to, respectively, be 40 and 1,000 orders of magnitude higher through the lenticels compared to the surrounding bark in *Betula potaninii* (Batalin) (Groh et al., 2002). Wittmann and Pfanz (2008a) measured bark conductance and stomatal conductance to water in a set of broadleaved tree species growing in conditions with high water availability and found that the stomatal conductance was 10 times higher than the bark conductance in high light conditions, but the ratio of stomatal to bark conductance decreased sharply with decreasing light availability. The results of Wittmann and Pfanz (2008a) imply that the role of water loss through bark in the whole-tree water use should be studied further in different species and environmental conditions.

In contrast to active stomatal control in leaves, the gas exchange through bark is passive and only regulated by long-term structural changes (Lendzian, 2006; Wittmann and Pfanz, 2008a) similarly to the gas exchange through leaf cuticles. Thus, although leaves are the dominant factor controlling seasonal transpiration and gas exchange, water loss through the bark before bud break has been estimated to be approximately 5% of total annual water loss in broadleaved species (Oren and Pataki, 2001). The role of water loss through the bark in the whole-tree water loss can be assumed to be proportionally large and physiologically important, especially when transpiration from foliage is at its minimum, as in springtime before bud burst in broadleaved species (Oren and Pataki, 2001), before springtime recovery of foliage photosynthesis in evergreen species (Peltoniemi et al., 2015) and during periods of drought (Klein et al., 2016; Wolfe, 2020). During drought, the stomata are closing, but water loss through the bark and leaf cuticle continues and may increase the risk of run-away cavitation leading to hydraulic failure (McDowell et al., 2013; Mencuccini et al., 2015). Thus, low cuticular and bark conductances have been suggested to be important determinants for tree drought survival (Santiago et al. 2016; Wolfe, 2020; Duursma et al. 2019).

When carbon assimilation in the leaves is minimised and/or phloem transport reduced (e.g., during drought), bark also plays an important role in tree carbon balance (De Roo et al., 2020). CO_2_ emissions through bark are determined mainly by the difference in branch respiration, bark photosynthesis, and axial transport of CO_2_ along with the xylem sap. Stem and branch respiration is an important component in the annual carbon balance of a forest and has been shown to contribute to 9% of total carbon loss from a boreal, *Pinus sylvestris* (L.)-dominated forest ecosystem (Zha et al., 2004). Stem and branch respiration is mainly driven by temperature (Sprugel, 1990) but is also affected by drought due to the reduction in metabolic activity and growth caused by low stem water status (Saveyn et al., 2007a; Saveyn et al., 2007b). Bark photosynthesis is mainly driven by the availability of photosynthetically active radiation (PAR) (e.g., De Roo et al., 2020), as the internal stem CO_2_ concentration is high (Teskey et al., 2008). The photosynthetic rate in woody tissues typically counterbalances 50–70% of stem dark respiration (Cernusak and Marshall, 2000; De Roo et al., 2020; Tarvainen et al., 2014; Wittmann and Pfanz, 2008b). Besides photosynthesis and respiration, the local stem CO_2_ emission is also affected by the xylem transpiration stream, as part of the CO_2_ locally released by respiration dissolves in the sap and moves upward in the transpiration stream in the xylem (Angert et al., 2012; Aubrey and Teskey, 2021; Bowman et al., 2005; McGuire and Teskey, 2004; Salomón et al., 2018; Salomón et al., 2019; Teskey and McGuire, 2007).

Not many attempts have been made to study transpiration through the bark in mature trees (Oren and Pataki, 2001; Wittman and Pfanz, 2008a; Wittman and Pfanz, 2008b; Wolfe, 2020) despite its potential importance for trees, especially in dry conditions. Here, we study the diurnal and seasonal dynamics and the role of branch water loss in *Pinus halepensis* (Mill.) growing in two contrasting plots (control and irrigated) in Israel. *Pinus halepensis* is well adapted to water stress but still has a narrow hydraulic safety margin of 0.3 MPa (from stomatal closure to 50% loss of hydraulic conductivity, Klein et al., 2011). We measured separately the transpiration rate from intact, leafless branch segments and transpiration from needle-bearing shoots (i.e., branch tips) to attempt to partition the water lost through the open stomata, leaf cuticle (i.e., minimum needle transpiration) and bark. In addition, we measured the CO_2_ emission rates from the leafless branch segments to quantify how drought affects the metabolic processes of the woody tissue. Measurements were carried out in two contrasting dry and irrigated plots to compare the effect of tree water status on bark transpiration and CO_2_ emission. We hypothesised that unlike leaf transpiration and bark CO_2_ efflux, bark transpiration is not affected by tree water status and may thus play a crucial role in total tree water use during the very dry summer conditions in the Yatir forest. Thus, we hypothesize that the role of bark water loss is high at the whole-tree level in drought conditions and/or when the stomata are closed.

## Materials and Methods

### Study Site and Manipulation Set-Up

The measurements were conducted at the Fluxnet Eddy covariance research site (Grünzweig et al., 2003; Rotenberg and Yakir, 2010) in a semi-arid Yatir forest, located at the edge of the Mediterranean region and at the northern edge of the Negev desert (31°20’N; 35°03′E; elevation 550–700 m a.s.l.). The Yatir forest has 200–250 biologically dry days annually. The forest site is a monoculture of *Pinus halepensis* and was planted during 1965–1969. The forest is sparse (~ 300 trees/ha and leaf area index approximately 1.7), tree height is *ca*. 11 m and mean stem diameter at breast height is 18 cm. The main active growing period is typically from January to April during the winter rains (Atzmon et al., 2004; Preisler et al., 2020). The forest soil is light Rendzina above chalk and limestone with an inaccessible groundwater table.

The forest site has annual mean global radiation of approximately 7.5 GJ m^−2^ y^−1^, mean annual precipitation of 280 mm, potential evapotranspiration of 1,600 mm year^−1^ and an annual mean temperature of 18.2°C. The site has an irrigation experiment, which includes an irrigated plot (1,000 m^2^) and a non-irrigated plot (control, 1,000 m^2^) with a buffer zone area in between. Irrigation began in May 2017. Soil moisture is kept constant (at relative soil water content ~33%) in the irrigated area and the drip irrigation is adjusted accordingly (e.g., ~850 mm of water was added by irrigation between May 2017 and January 2018), whereas it changes seasonally in the control area (from relative soil water content 35 to 8%).

### Gas Exchange Measurements

**Figure 1:**
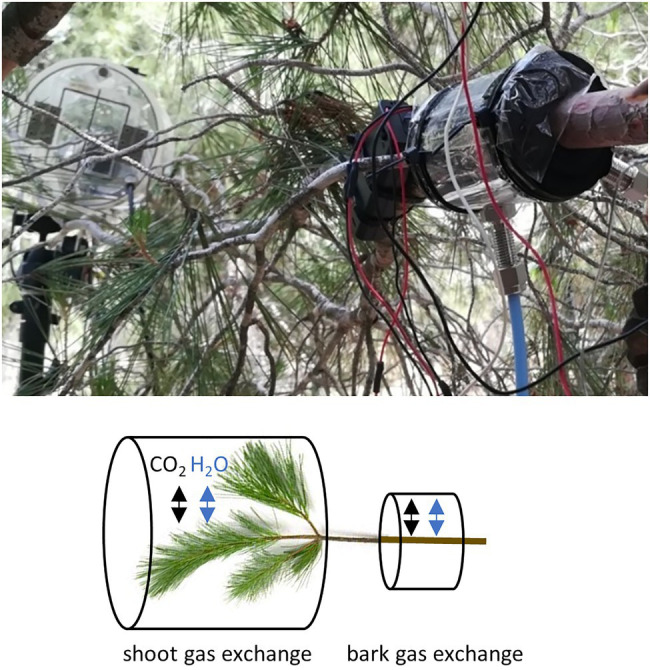
A photograph and a schematic presentation of the gas exchange measurement set-up in a branch. From each intact sample branch, we measured the exchange of water and CO_2_ through bark from a branch segment without any needles and from a needle-bearing shoot.

We measured H_2_O and CO_2_ exchange of *Pinus halepensis* branches from trees growing at the study site from June to November 2019. We measured one branch from altogether 10 trees, 5 at each plot. From each measured branch, we simultaneously measured gas exchange through bark from a leafless branch segment and a shoot (i.e., branch tip) with needles (Figure 1). The average diameter of the measured leafless branch segments was 26 ± 4 mm. The cuvette system allowed measuring two branches simultaneously (Table 1), one branch from the control and one from the irrigated plot.

**Table 1:** The cuvette design allowed simultaneous gas exchange measurements of two trees, i.e., a tree pair consisting of one irrigated tree and one control tree.

Tree pair: consists of 1 control and 1 irrigated tree	Measurement days in year 2019
1	June 6th to 10th
2	June 10th to 12th
3	June 12th to 17th
4	June 17th to 19th
5	June 20th to November 30th

*The study design consisted of all together 5 tree pairs. The exact measurement days are shown in this table. The aim with this study design was to obtain measurements from five repetitions of tree pairs, and in addition, a good temporal coverage with the last pair of trees*.

Gas exchange per bark area was measured with a cuvette (outside length 10 cm, inside length 5.5 cm, diameter varied with branch size and mounting, average air volume inside cuvette 111 cm^3^) made of transparent polyethylene plastic, wrapped around the central part of the branch, sealed at both ends with foam and tightened with cable ties (Figure 1). The joint of the cuvette was sealed with transparent tape. To allow constant air circulation, gas inlet and outlet tubes were connected to the cuvette and a battery-powered fan (1 lpm) inserted into the cuvette. The gas exchange of the sample was measured with a mobile H_2_O and CO_2_ open-flow cuvette system including a high-resolution LI-840A gas analyser (LI-COR, Nebraska, United States). Raspberry Pi was utilised to communicate with the LI-840A, Nokeval 8-channel transmitter (Nokeval, Nokia, Finland) and user-defined measuring protocols. Ambient air was continuously passed through the cuvettes at a constant rate, and measurements were based on the concentration of the air stream flowing out of the cuvette and a reference airline. The air stream concentration was measured from both cuvettes every three minutes. See details of the measurement system in Lintunen et al. (2020). Surface area of the bark was calculated based on branch diameter measurement assuming a cylinder branch shape inside the cuvette with 5.5 cm inner length.

Gas exchange from needle-bearing shoots was measured continuously from June to November 2019 from the same 10 trees with automatic shoot cuvettes. The cuvettes were cylindrical in shape, transparent and open from one end most of the time to allow the shoot to interact with the ambient air. About once an hour the lid of each cuvette closed for four minutes and air within the cuvette was pumped (10 l min^−1^) through the sample cell of a centrally located infra-red gas analyser (Li-7,000, Licor, Lincoln NE, United States), while a similar flow of ambient air in the canopy was pumped through the reference cell of the analyser to obtain a differential concentration measurements of CO_2_ and H_2_O concentrations (see Preisler Y., 2019, PhD Thesis, Weizmann Institute of Science). Surface area of a shoot was calculated as the sum of bark area and projected needle area to account for the mutual shading effect between the needles. This was done by using image analysis to estimate relative shaded area created by four twigs as they arranged randomly in the cuvette. This relative shading was used to convert total needle area to projected needle area (Oz I, 2021 MSc. Thesis, Weizmann Institute of Science, in revisions).

Water condensation inside the cuvettes and the tubing is often a challenge when measuring gas exchange during high air humidity conditions. At our site, the temperature decreased close to dew point only during some nights. Nights with water condensation were followed by high momentary peaks in bark transpiration during early mornings. Such mornings were identified from the data by having momentary bark transpiration rates higher than 0.33 mmol m^−2^ s^−1^ before or at 9 am. There were two nights with water condensation in June, nine in July, 14 in August, 18 in September, 15 in October and three in November. Days with water condensation in the system were eliminated from the data to avoid measurement error. In addition to these eliminated full days, hourly values with high risk for dew formation in the shoot cuvettes (identified by monitoring an empty shoot cuvette) and values below the accuracy of the Li-7,000 analyser were eliminated from the shoot gas exchange data.

### Other On-Site Measurements

**Figure 2:**
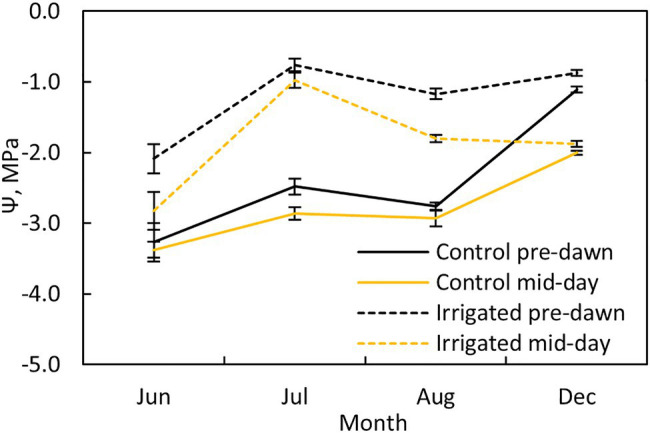
Tree water potential at pre-dawn (black lines) and midday (yellow lines) during different months at the control (solid line) and irrigated plots (dashed line). Error bars show standard error (*n* = 6).

Pre-dawn and midday leaf water potentials were measured at both plots once a month (Figure 2; four times during the measurement period) using a pressure chamber (PMS, Albany, United States). Two twigs were sampled from two sides of the tree (north and south) from three trees at each plot (n = 6 per plot).

Branch temperature was measured continuously starting from 10th of June with a PT100 (Pentronic, Sweden) temperature sensor attached into the surface of one branch at the stand, similar to the ones measured with the cuvettes.

Meteorological variables, including air temperature and air humidity, were provided by the local Yatir Flux Tower site (http://fluxnet.ornl.gov/site/522, Qubaja et al., 2020a; Qubaja et al., 2020b). Photosynthetic active radiation was measured from one location in the middle of the stand at canopy height (PQS 1, Kipp & Zonen, Delft, Netherlands).

The driving force for bark transpiration was calculated from the meteorological variables as the modelled bark transpiration rate (*E_0_*). *E_0_* (mmol m^−2^ s^−1^) was calculated to be the difference between the water vapour concentration (kPa) inside the branch (*w_i,0_*) and water vapour concentration (kPa) in the ambient air (*w_a_*).


$$E_{0}=\left(w_{i,0}-w_{a}\right)g,$$ (1)

where *g* is bark conductance (assumed to be constant). Note that *Wi,_0_ – W_a_* is not the same as the commonly used vapour pressure deficit (VPD), as air and branch temperatures are not typically equal to each other. We calculated the water vapour concentration inside the branch from the branch temperature assuming that the vapour pressure inside the branch was saturated. In reality, the vapour pressure in the branch is slightly lower than the saturation vapour pressure due to the effect of the lower water potential (Vesala et al., 2017). However, as we did not have continuous data on branch water potential and the effect of the lowered water potential is rather small (Vesala et al., 2017), we neglected its effect in the calculation of the evaporative driving force.

### Statistical Analysis

**Figure 3:**
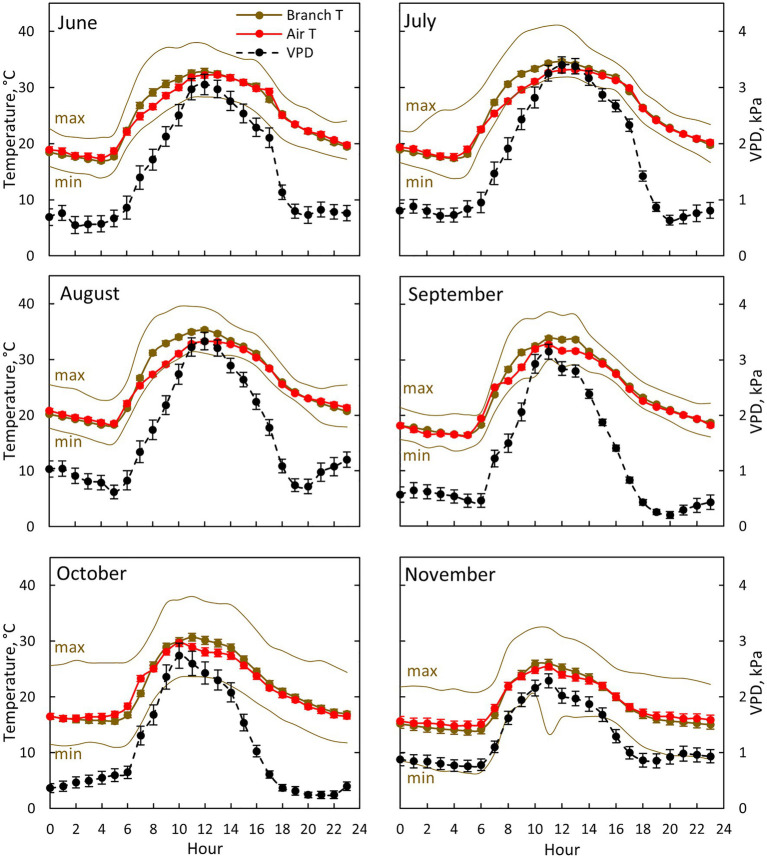
Air (red colour) and branch (brown colour) temperature together with ambient vapour pressure deficit (VPD, black colour) for each month between June (10th of June onwards) and November in 2019. Each subfigure represents hourly averages for one month. For branch temperature, also hourly average temperature for the warmest (max) and coldest (min) day for each month is shown. Error bars show standard error (hourly averages are first calculated for each day and then averaged for the month).

**Figure 4:**
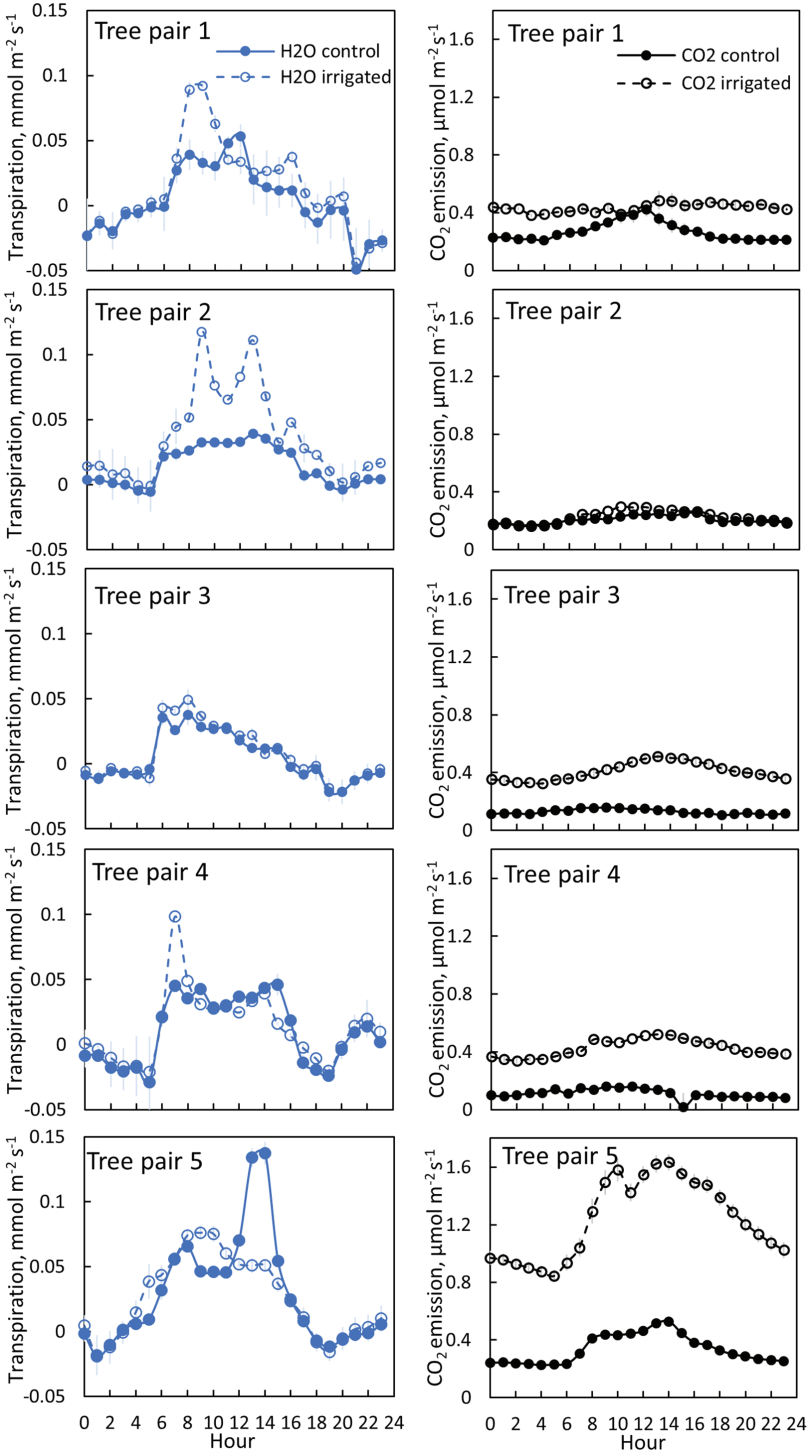
Bark transpiration (blue symbols) and CO_2_ emission (black symbols) in a control (filled symbols) and irrigated tree (empty symbols) in June 2019. The first pair of branches was measured from 6th to 10thof June, the second pair from 10th to 12th of June, the third pair from 12th to 17th of June, the fourth pair from 17th to 19th of June and the fifth pair from 20th of June to 30th of June. Each subfigure represents hourly averages for one pair of trees measured simultaneously. Error bars show standard error (hourly averages are first calculated for each day and then averaged for the measurement period).

**Figure 5:**
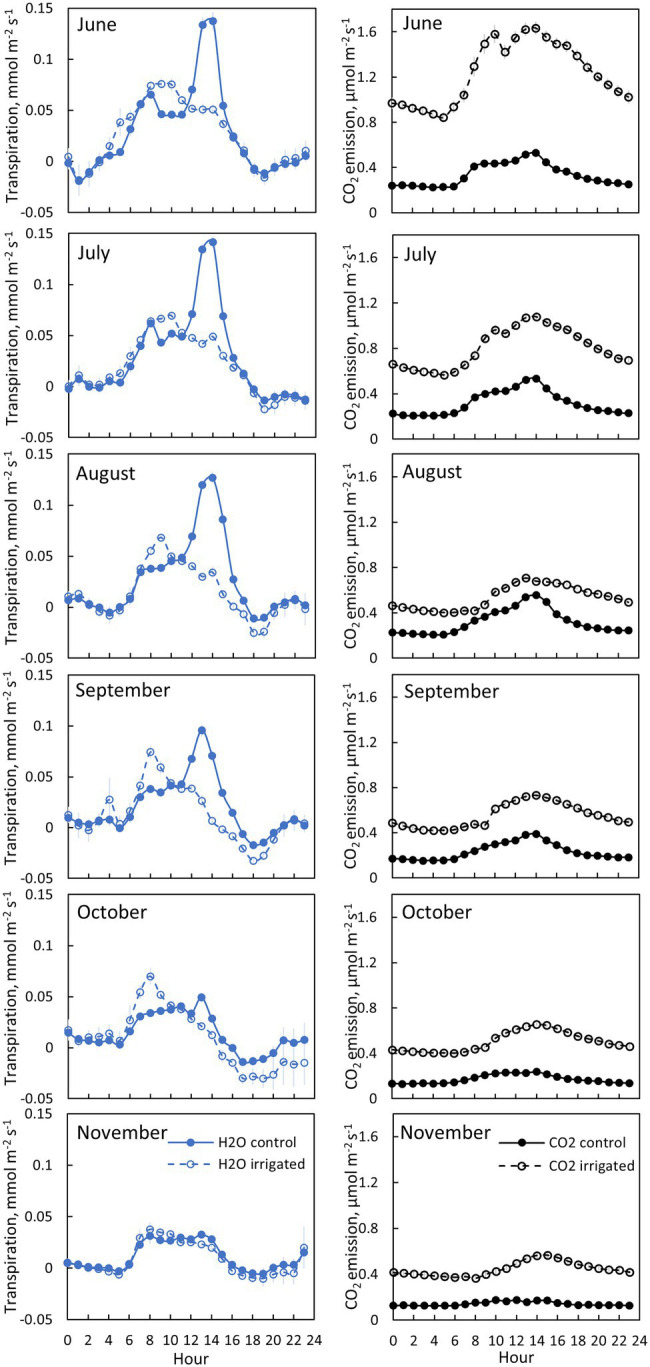
Bark transpiration (blue symbols) and CO_2_ emission (black symbols) in a control (filled symbols) and an irrigated tree (empty symbols) in tree pair 5 from 20th of June to 30th of November 2019. Each subfigure represents hourly averages for one month. Error bars show standard error (hourly averages are first calculated for each day and then averaged for the month).

The gas exchange results and environmental conditions are mainly presented as hourly means. To analyse the daily dynamics of environmental conditions, bark transpiration, and CO_2_ exchange, means and standard errors of hourly data are calculated for each measured tree pair and month (in Figures 3–5). To compare water loss from branches between the plots, we used Tukey–Kramer analysis in proc. GLM (SAS version 9.4, SAS Institute Inc., Cary, NC). To analyse linear regression between bark transpiration and evaporative driving force (Eq. 1) and exponential regression between CO_2_ exchange through bark and branch temperature in selected time periods in June and August, we used proc. NLIN that can fit nonlinear regression models and estimates the parameters by nonlinear least squares (SAS version 9.4, SAS Institute Inc., Cary, NC). The difference between two variables was considered statistically significant if the 95% confidence interval (95%CI) of the regression parameter was not overlapping.

## Results

### Water Potential, Branch Temperature and Ambient Conditions

Water potential at pre-dawn and midday was ~1.5 MPa higher in the irrigated plot compared to the control plot during the dry summer months (Figure 2). The stomata were nearly closed in control plot trees during the dry months, as indicated by the small difference between the pre-dawn and midday water potentials at this plot, whereas they were more open in the irrigated plot trees (except in July).

Air temperature, branch temperature and ambient VPD were the highest at midday from July to September (Figure 3). Branch temperature was in equilibrium with air temperature during the evening, night and early morning, whereas the branch was warmer than air during the day (Figure 3). The difference was at its maximum (3.9°C) at 8:00–9:00 in August, whereas the difference was negligible in November. Ambient VPD was at its maximum at noon in July and August (3.3–3.4 kPa) and at its minimum (0.2 kPa) during the nights in September and October.

### Transpiration Through Bark

**Figure 6:**
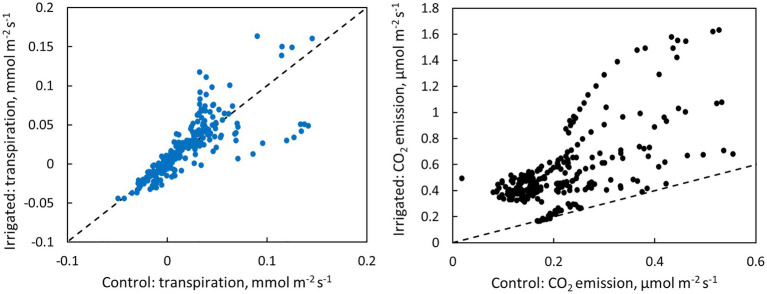
Scatter plots of bark transpiration and CO_2_ emission between the control and irrigated plots. Each circle represents hourly averages of one measured tree pair in June (data from Figure 4) or one measured month of tree pair 5 in July–November (data from Figure 5). The dashed line represents the 1:1 line.

Transpiration through bark was observed from all measured branches and for all measured months (Figures 4–5, Table 2). The maximum daily bark transpiration rate was 0.03–0.14 mmol m^−2^ s^−1^ depending on the tree and the measurement month. Bark transpiration dynamics followed the general trend where transpiration was low during the night and in the evening, and higher during the early morning and day. The timing of the transpiration maximum(s) varied between tree individuals and months due to the different light environments affecting the branch temperature. Also, negative values of transpiration (i.e., water uptake) were observed during night time in all the trees (Figures 4, 5). The highest night time negative transpiration rate of 0.05 mmol m^−2^ s^−1^ was measured in tree pair 1 during June (Figure 4). Bark transpiration was similar between the treatments (Figure 6), although occasionally either the irrigated branches (in tree pairs 1–4) or control branches (in tree pair 5) showed higher maximum bark transpiration rates during the day (Figures 4, 5). This was related to different solar radiation conditions of the branches: in tree pairs 1–4, the irrigated branches received more sun light during the day than the control branches, whereas the opposite was true for tree pair 5 (see Supplementary Material). The average diurnal bark transpiration rate for the measured trees in June was 0.010 (SE 0.004) mmol m^−2^ s^−1^ in the control plot and 0.016 (SE 0.004) mmol m^−2^ s^−1^ in the irrigated plot. The difference between the plots was not statistically significant (*p* = 0.3780, R^2^ = 0.098, *F*-value = 0.87). Bark transpiration decreased from June to November in both the control and irrigated tree of pair 5 (Figure 5).

**Table 2:** Mean day and night time bark transpiration and CO_2_ exchange (± standard error) for the measured tree pairs and months. The means are calculated from hourly averages.

Tree pair	Treatment	Month	Day/night	Transpiration (mmol m^−2^ s^−1^)	CO_2_ exchange (μmol m^−2^ s^−1^)
1	Control	6	Day	0.021 (±0.004)	0.309 (±0.013)
1	Control	6	Night	−0.017 (±0.004)	0.220 (±0.004)
1	Irrigation	6	Day	0.038 (±0.006)	0.439 (±0.009)
1	Irrigation	6	Night	−0.014 (±0.005)	0.427 (±0.006)
2	Control	6	Day	0.024 (±0.003)	0.220 (±0.006)
2	Control	6	Night	0.000 (±0.002)	0.184 (±0.004)
2	Irrigation	6	Day	0.053 (±0.007)	0.255 (±0.006)
2	Irrigation	6	Night	0.008 (±0.003)	0.188 (±0.004)
3	Control	6	Day	0.017 (±0.002)	0.137 (±0.003)
3	Control	6	Night	−0.011 (±0.001)	0.118 (±0.002)
3	Irrigation	6	Day	0.021 (±0.002)	0.450 (±0.009)
3	Irrigation	6	Night	−0.010 (±0.002)	0.361 (±0.007)
4	Control	6	Day	0.022 (±0.006)	0.110 (±0.013)
4	Control	6	Night	−0.009 (±0.004)	0.100 (±0.004)
4	Irrigation	6	Day	0.022 (±0.006)	0.468 (±0.010)
4	Irrigation	6	Night	−0.004 (±0.004)	0.374 (±0.007)
5	Control	6	Day	0.055 (±0.004)	0.406 (±0.010)
5	Control	6	Night	−0.003 (±0.002)	0.252 (±0.004)
5	Irrigation	6	Day	0.046 (±0.003)	1.422 (±0.025)
5	Irrigation	6	Night	0.002 (±0.003)	1.017 (±0.019)
5	Control	7	Day	0.055 (±0.003)	0.392 (±0.007)
5	Control	7	Night	−0.004 (±0.002)	0.228 (±0.002)
5	Irrigation	7	Day	0.040 (±0.002)	0.909 (±0.014)
5	Irrigation	7	Night	−0.003 (±0.002)	0.678 (±0.010)
5	Control	8	Day	0.049 (±0.003)	0.393 (±0.007)
5	Control	8	Night	0.001 (±0.002)	0.231 (±0.003)
5	Irrigation	8	Day	0.027 (±0.002)	0.582 (±0.008)
5	Irrigation	8	Night	−0.001 (±0.002)	0.474 (±0.006)
5	Control	9	Day	0.035 (±0.003)	0.284 (±0.006)
5	Control	9	Night	0.002 (±0.002)	0.172 (±0.003)
5	Irrigation	9	Day	0.022 (±0.003)	0.608 (±0.011)
5	Irrigation	9	Night	0.003 (±0.004)	0.480 (±0.007)
5	Control	10	Day	0.022 (±0.002)	0.200 (±0.003)
5	Control	10	Night	0.004 (±0.002)	0.140 (±0.002)
5	Irrigation	10	Day	0.020 (±0.003)	0.548 (±0.007)
5	Irrigation	10	Night	−0.003 (±0.004)	0.448 (±0.006)
5	Control	11	Day	0.019 (±0.001)	0.155 (±0.002)
5	Control	11	Night	0.002 (±0.002)	0.129 (±0.001)
5	Irrigation	11	Day	0.017 (±0.001)	0.469 (±0.008)
5	Irrigation	11	Night	−0.001 (±0.003)	0.418 (±0.006)

**Figure 7:**
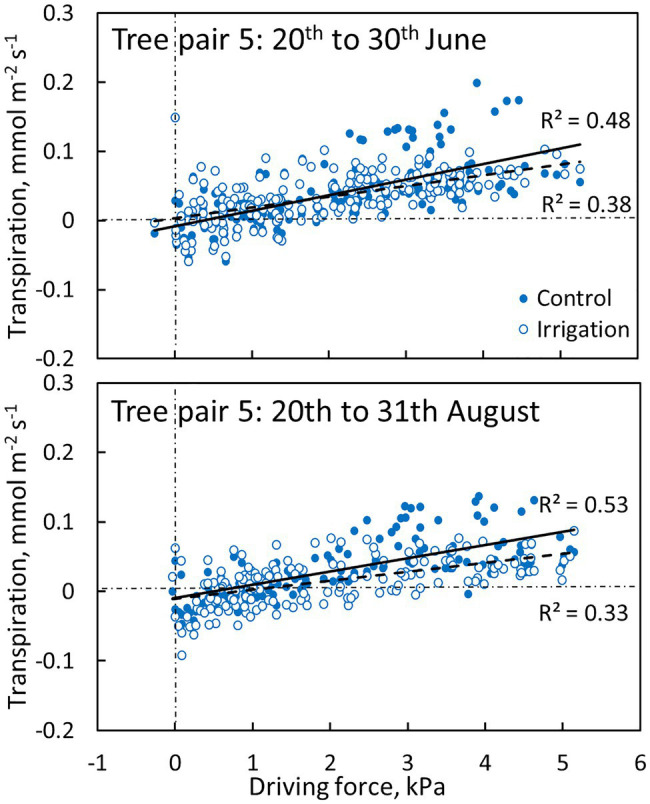
The relation between transpiration through bark and evaporative driving force (Eq. 1) in a control (filled symbols and solid regression line) and irrigated tree (empty symbols and dashed regression line) in tree pair 5 over a 10-day period in June and August 2019. Values are hourly values. Linear fits are shown (*p* < 0.0001).

Transpiration through the bark was dependent on the driving force for transpiration (w_i,0_-w_a_, see Eq. 1). The linear regression between transpiration and evaporative driving force was slightly higher in the control plot branch compared to the irrigated plot branch in June and August (Figure 7, Table 3). Water uptake by the branches occurred during certain nights when air humidity was high (and driving force low, Figure 7).

**Table 3:** Statistical analysis results for Figures 7 and 8: Linear regression between bark transpiration (mmol m^−2^ s^−1^) and evaporative driving force (kPa, Eq. 1), and exponential regression between CO_2_ exchange through bark (μmol m^−2^ s^−1^) and branch temperature (°C) on 20th to 30th of June and 20th to 31st of August.

Treatment	Month	Day/Night	a	95% CI	b	95% CI	P
Bark transpiration = a^*^Driver + b							
Control	June	NA	0.023	0.019–0.026	−0.009	−0.016-(−0.001)	<0.0001
Irrigated	June	NA	0.016	0.013–0.018	0.003	-0.003-0.009	<0.0001
Control	August	NA	0.019	0.017–0.022	−0.010	−0.016-(−0.003)	<0.0001
Irrigated	August	NA	0.013	0.010−0.016	−0.010	−0.017-(−0.004)	<0.0001
CO2 emission = a^*^exp(b^*^branchT)							
Control	June	Day	0.050	0.035–0.066	0.066	0.057–0.075	<0.0001
Control	June	Night	0.101	0.088–0.113	0.046	0.040–0.052	<0.0001
Irrigated	June	Day	0.354	0.269–0.439	0.044	0.037–0.051	<0.0001
Irrigated	June	Night	0.364	0.318–0.410	0.052	0.046–0.057	<0.0001
Control	August	Day	0.077	0.053–0.101	0.048	0.039–0.057	<0.0001
Control	August	Night	0.080	0.071–0.088	0.047	0.042–0.052	<0.0001
Irrigated	August	Day	0.212	0.152–0.271	0.032	0.023–0.040	<0.0001
Irrigated	August	Night	0.169	0.155–0.183	0.051	0.047–0.055	<0.0001

*For the CO_2_ emission, analysis was done separately for daytime (6:00–18:00) and night time (18:00–6:00)*.

### CO_2_ Emission Through Bark

CO_2_ emission through the bark was always higher in irrigated than in control trees (Figure 6). The daily maximum CO_2_ emission rate was 0.2–0.6 μmol m^−2^ s^−1^ at the control plot and 0.3–1.6 μmol m^−2^ s^−1^ at the irrigated plot depending on the tree and measurement month (Figures 4, 5). The daily maximum CO_2_ emission rate occurred typically in the afternoon, later than the maximum bark transpiration rate. CO_2_ emission measured from the irrigated tree decreased strongly from June to August (Figure 5), and particularly the trend of high CO_2_ emission during the day weakened from June to August and further until November (Figure 5). From August to November, CO_2_ emission from both the control and irrigated tree decreased slightly (Figure 5). The CO_2_ emission difference between the irrigated and the control tree was thus highest in June and July (Figure 5).

**Figure 8:**
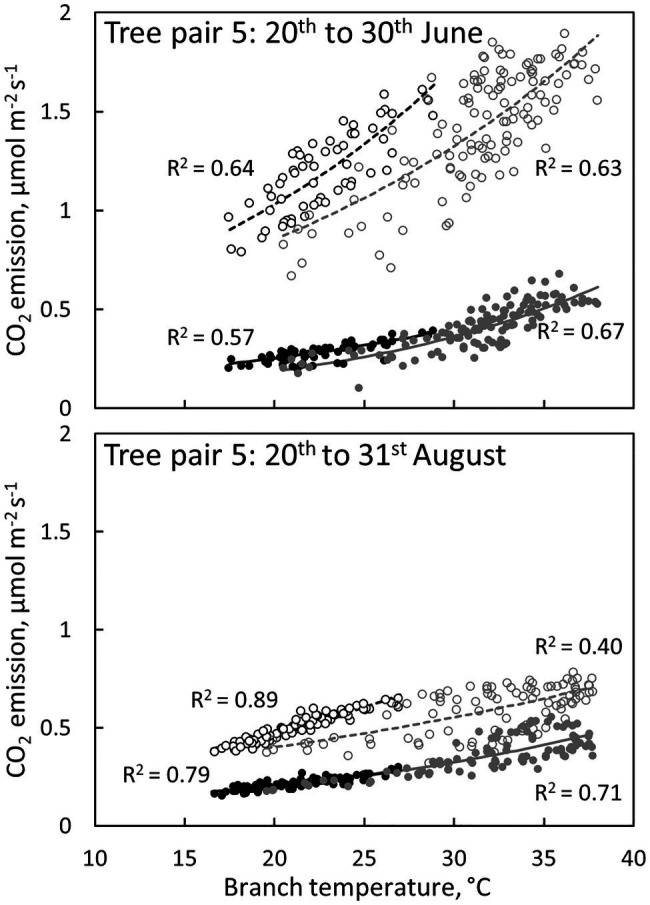
The relation between CO_2_ emission through the bark and branch temperature during the day (6:00–18:00, grey symbols and regression lines) and night (18:00–6:00, black symbols and regression lines) in a control tree (filled symbols and solid regression lines) and an irrigated tree (empty symbols and dashed regression lines) in tree pair 5 over a 10-day period in June and August 2019. Values are hourly values. Exponential fits are shown (p < 0.0001).

Branch CO_2_ emission was dependent on stem temperature, but it was higher in irrigated than in control trees for a given temperature (Figure 8, Table 3). This difference was higher in June and July than in other months (Figures 5, 8, Table 3), suggesting that the difference observed in CO_2_ emission between months is not explained solely by temperature. Branch CO_2_ emission for a given temperature was also higher during nights than during days in the irrigated plot, whereas we observed no clear difference between nights and days in the control plot (Figure 8, Table 3).

### Comparison of Bark Transpiration and Conductance With Shoot Transpiration and Conductance

**Figure 9:**
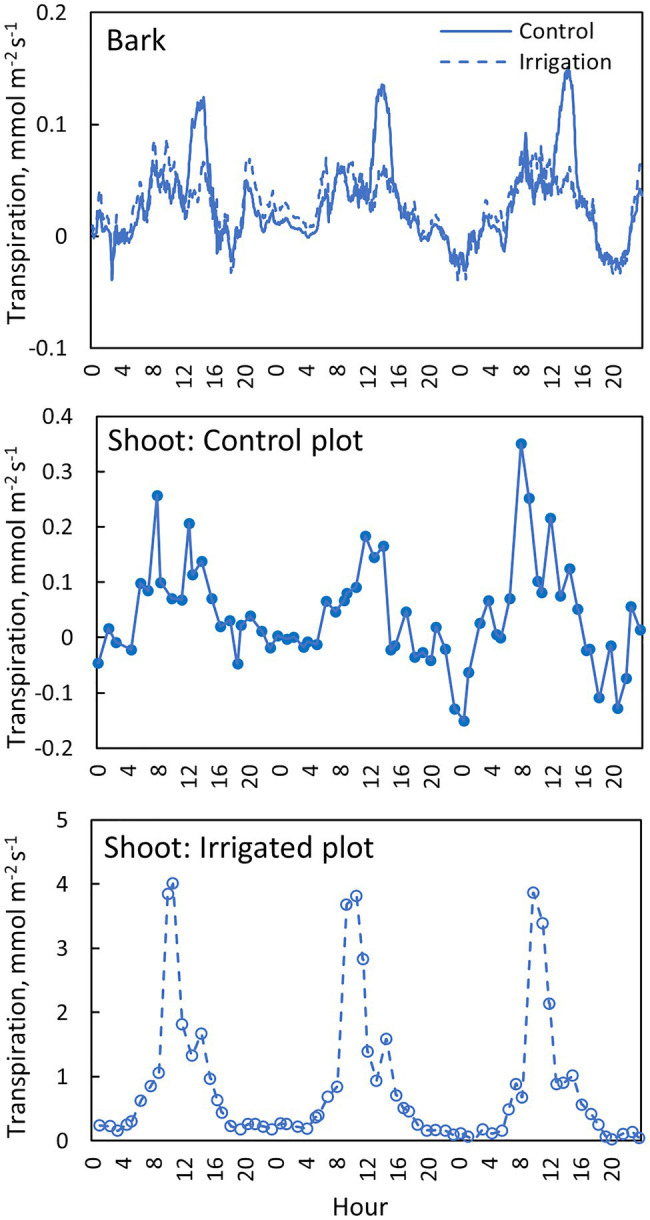
Transpiration through the bark in the needle-less branch segments (top figure), transpiration from the needle-bearing shoots in a control tree (middle figure) and an irrigated tree (bottom figure) in tree pair 5 from 1st to 3rd of July 2019. Transpiration through the bark is shown with a moving average of 10 observations (corresponds to 30 min).

**Figure 10:**
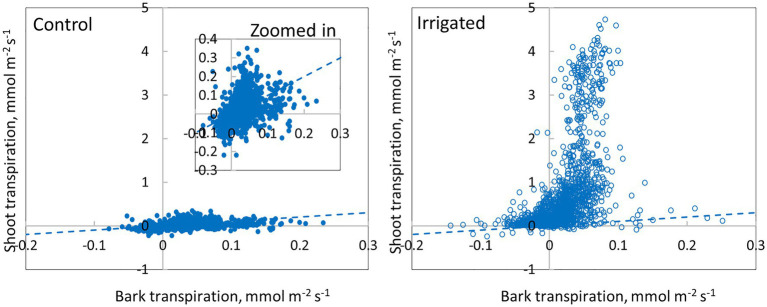
Comparison of bark transpiration (needle-less branch segments) and shoot transpiration (with needles) in a control and an irrigated tree (tree pair 5) from June to November 2019. Values are hourly values. The 1:1 line is drawn.

Daily dynamics of bark transpiration rate and shoot transpiration rate were rather similar, although the absolute water lost rates were different (Figure 9). The hourly average bark transpiration rate per bark surface area was 76% of the hourly average shoot transpiration rate per shoot surface area in the control trees that showed only minimum needle transpiration (Figure 10). Especially in conditions with high evaporative demand in the control plot, bark transpiration rate seemed to even exceed shoot transpiration rate, whereas shoot transpiration rate was higher in conditions with low evaporative demand (Figure 10). In the irrigated trees with active stomatal control, an average transpiration rate per shoot area was 27-fold compared to control trees and 36-fold compared to the bark transpiration rate (which was of the same size in the irrigated and control trees, Figure 10).

Mean total bark and shoot conductance for water vapour in the control and irrigated plot were calculated for daytime (10:00–16:00) in June (Table 4). Bark conductance in the control plot was 1.96 mmol m^−2^ s^−1^ and shoot conductance in the control plot was 2.60 mmol m^−2^ s^−1^. Thus, in accordance with the transpiration results, bark conductance was 75% of the shoot conductance in the control plot. In the irrigated plot with active stomatal control in the shoot, bark conductance was 1.46 mmol m^−2^ s^−1^, whereas shoot conductance was 50.7 mmol m^−2^ s^−1^.

**Table 4:** Total bark and shoot conductance (g) to water vapour in control and irrigated plot. Values are hourly means measured between 10:00 and 16:00 in June.

Organ	Treatment	g (mmol m^−2^ s^−1^)
bark	control	1.96 (±0.13)
bark	irrigated	1.46 (±0.07)
shoot	control	2.60 (±0.30)
shoot	irrigated	50.7 (±4.27)

*Here, the driving force (* $w_{i,0}-w_{a,}\;Eg.1$ *) was expressed in unitless form as the difference between the molar concentration of water vapour inside the branch and the molar concentration of water vapour in the ambient air as typically done (Wittmann* et al. *2006**). Also standard error is shown (hourly averages of measured trees are first calculated for each day and then averaged)*.

## Discussion

### The Role of Bark Transpiration in the Whole-Tree Water Loss

Our results demonstrate that water loss through the bark plays an important role in the whole-tree water balance of *Pinus halepensis* during drought periods, and especially during conditions with high evaporative demand. Bark transpiration rate measured from a needle-less branch segment was typically 0.05–0.10 mmol m^−2^ s^−1^ during the day (Figures 4, 5) but could momentarily reach 0.20 mmol m^−2^ s^−1^ (Figure 10). The passive water loss rate through the bark was not affected by drought, unlike the water loss rate from the needle-bearing shoots, which was strongly affected by drought due to active stomata functioning in needles. Thus, the ratio of hourly water loss rate from the shoots to water loss rate through the bark averaged 36 times higher in the irrigated plot compared with the drought-stressed control plot (calculated based on Figure 10). The transpiration rates through the bark based on surface area were typically lower than the observed values in shoot transpiration rates in control trees (with minimum needle transpiration) when VPD was low and exceeded these values when VPD was high (Figure 10). The role of transpiration through bark should thus be taken into account as part of minimum conductance in models of water use efficiency and drought responses.

The rate of bark transpiration for *Pinus halepensis* measured in this study was rather similar to the rates measured for broadleaved tree species by Wittmann and Pfanz (2008a). They measured a bark transpiration rate between 0.08 and 0.28 mmol m^−2^ s^−1^ at an air temperature of 20°C and a relative humidity of 45–50% from well-irrigated, young *Betula pendula* (Roth), *Fagus sylvatica* (L.), *Quercus robur* (L.), *Alnus glutinosa* ((L.) Gaertn.) and *Prunus avium* (L.) current-year stems 2–6 mm in diameter. On contrary to the rate of transpiration through bark, the daytime transpiration of shoots was 70–90% lower in *Pinus halepensis* at the control plot of this study (i.e., needles with presumed nearly closed stomata) than in the leaves of the five broadleaved tree species measured by Wittmann and Pfanz (2008a). Also this demonstrates that although active needle transpiration strongly adjusts to scarce water availability, passive transpiration through bark does not. *Pinus halepensis* is a drought-adapted conifer that avoids drought and saves water by closing stomata and keeping a narrow hydraulic safety margin. It is known to drastically reduce its water use during drought through stomatal closure (Borghetti et al., 1998; Ferrio et al., 2003; Klein et al., 2011; Melzack et al., 1985) and by decreasing its growth until water becomes available again (Ferrio et al., 2003; Girard et al., 2012; Liphschitz and Lev-Yadun, 1986; Nicault et al., 2001). Atzmon et al. (2004) measured the daytime shoot transpiration of *Pinus halepensis* at the same site to be ~0.4 mmol m^−2^ s^−1^ for the winter months in 1998–1999. Accordingly, we measured daytime shoot transpiration to be 0.1–0.3 mmol m^−2^ s^−1^ for the control trees during dry summer and autumn months (June to November) in 2019. Also Maseyk et al. (2008) made transpiration measurements at the same site in 2000–2005 and recorded higher transpiration rates for the summer months, around ~0.5 mmol m^−2^ s^−1^. This difference can be partially explained by different methods used as Maseyk et al. (2008) used needle-scale cuvette for the gas exchange measurements, whereas we used here shoot-scale cuvette for the gas exchange measurements.

Bark conductance of different species and trees growing in different conditions is not well known. Wolfe (2020) measured bark conductance in saplings of eight tropical tree species in drought conditions and found a positive correlation between bark conductance and stem water deficit, suggesting that high bark conductance for water vapour can indeed cause stem water deficit. The bark conductance measured in our study for drought-adapted *Pinus halepensis* (1.46–1.96 mmol m^−2^ s^−1^) is in the lower range of the bark conductances measured by Wolfe (2020) for tropical tree species (0.86 to 12.98 mmol m^−2^ s^−1^) and by Wittmann and Pfanz (2008a) for temperate angiosperm species (5.01–27.3 mmol m^−2^ s^−1^), but similar to measurements by Cernusak and Marshall (2000) for another *Pinus*-species, *Pinus monticola* (1.03 mmol m^−2^ s^−1^). These previously reported values for bark conductance are rather similar to values of minimum leaf conductance (4.89 ± 2.67 mmol m^−2^ s^−1^) reported by Duursma et al. (2019) in a meta-analysis. Similarly, the bark conductance of *Pinus halepensis* measured in this study was 75% of the shoot conductance in the control plot, where needles can be assumed to have nearly closed stomata, and 3% in the irrigated plot, where stomata in the needles are more open.

There are also some studies linking bark structural properties to bark water dynamics (see Van Stan et al. 2021). Loram-Lourenço et al. (2020) studied the bark properties of 31 native tree species from Brazil and found that the relative investment in bark reflects different strategies of fire protection, and water use and conservation. They found that species with a thicker and less dense inner bark had the highest water contents in the wood, bark and leaves. They concluded that inner bark properties were associated with the regulation of water status and photosynthetic capacity, and outer bark properties with defence against pathogen attack, mechanical support and fire resistance. Similarly, Martín-Sanz et al. (2019) showed that *Pinus halepensis* trees growing at moister sites allocated more resources to the bark, whereas trees growing at drier sites allocated fewer resources to the bark, resulting in thinner bark. The supplement summer irrigation began in 2017, so no genetic adaptations are possible between the studied trees at our two contrasting plots, and thus, any possible differences in the bark characteristics of the control and irrigated trees would be caused by acclimation to the prevailing environmental conditions. Our results suggest that there were no major differences in the bark characteristics (for example lenticel density) between the plots, i.e., no structural acclimation to irrigation two years after irrigation commenced. The small (but not statistically significant) difference found in the average transpiration through bark between the plots could very likely be explained by difference in individual branch temperatures caused by varying local radiation conditions (see Fig. S1 in the Supplementary Material) as the difference in the average transpiration through bark was visible daily mainly during the time of maximum transpiration. As branch temperature was only measured from one branch at the middle of the site, the possible differences in branch temperatures caused by varying local radiation conditions could not be considered in the analysis of the evaporative driving force (Figure 7). Lacking measurements of branch and air temperatures inside the branch cuvettes (we used branch and air temperatures measured outside a cuvette) also cause small underestimation (~3%, see Figures S2-S4 in the Supplementary Material) to the driving force for bark transpiration (Figure 7), because the cuvettes warm up during day time due to the greenhouse effect. This further leads to small overestimation of bark (and shoot) conductance.

**Figure 11:**
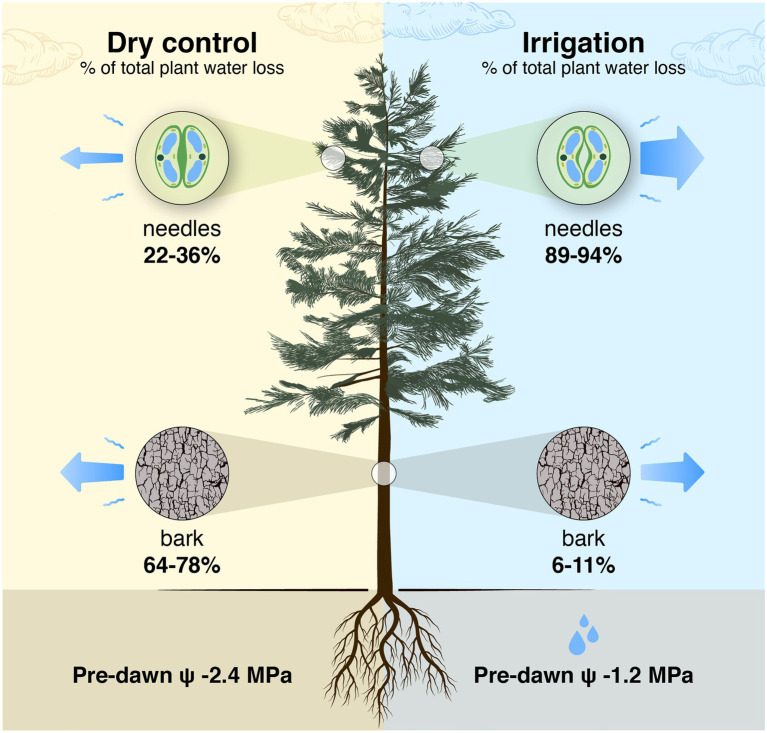
Schematic presentation of a tree-level estimation of the share of needles and bark in the total daily tree water loss in a control tree with closed stomata in a semi-arid environment and an irrigated tree with open stomata. The estimation is based on relative ratios of tree-level total needle and bark surface areas [2/1 to 1/1 based on Whittaker and Woodwell (1967), Lintunen et al. (2011) and Hölttä et al. (2013)] and area-based evapotranspiration rates of bark and needles measured in this study (data from Figure 10; shoot transpiration was partitioned into bark and needle transpiration based on bark surface area measurements). Shoot transpiration per projected needle area was converted to transpiration per total needle area by dividing it with 4.67 that is the ratio of total to projected needle area measured in a subsample of shoot cuvettes. The pre-dawn water potential (ψ) is the average of the measurements in Figure 2.

In young trees, bark surface area (including branches and trunk) is typically 1/3 and foliage area 2/3 of the aboveground plant surface area (Whittaker and Woodwell, 1967), which we assume also applies to *Pinus halepensis*. The ratio between bark area and foliage area increases with increasing tree size (Hölttä et al., 2013; West et al., 1997), and, e.g., in a 16-m tall *Pinus sylvestris* tree, the bark surface area can be 1/2 of the aboveground plant surface area (result based on models in Lintunen et al., 2011 and Hölttä et al., 2013). This suggests that the role of transpiration through bark is important to whole-tree water balance and will increase with tree size. As average bark transpiration rate was ~24% lower than the shoot transpiration rate in the control plot, bark transpiration is estimated to be responsible for 64–78% of total water loss during drought stress in *Pinus halepensis* trees (estimated based on relative ratios of surface areas and area-based transpiration rates of bark and needles, Figure 11). During high evaporative demand, the portion of bark transpiration is even higher in drought-stressed *Pinus halepensis* trees with closed stomata. In the irrigated trees, bark transpiration is responsible for 6–11% of total water loss (due to high transpiration through open stomata, Figure 11). Thus, the implications of transpiration through bark are of especially high importance when upscaling leaf-scale data to canopy transpiration in dry growth conditions where trees save water by minimising stomatal conductance.

### Bark Water Uptake During Most Summer Nights

Bark absorbed moisture from the air during most summer nights. The night time water uptake rate was typically ~40% of the daytime transpiration rate through the bark. We are not aware of any other studies, where water uptake by intact branches was evident in field conditions. Our results are in line with previous studies that show mass uptake of water by detached branches soaked in water or exposed to fully saturated air (Earles et al., 2016; Katz et al., 1989; Laur and Hacke, 2014; Liu et al., 2019). But does the water taken by the bark move beyond the bark into the xylem? Earles et al. (2016) showed with δ^18^O labelled water that water permeated the bark into the xylem of excised branch segments of *Sequoia sempervirens* ((D.Don) Endl.) soaked in water. They also demonstrated, using μCT images, that this resulted in a decreased embolized area. Similarly, Katz et al. (1989) showed that dye sprayed on the bark was later found in the stem parenchyma rays of *Picea glauca* ((Moench) Voss). Earles et al. 2016 have suggested a symplastic role in transferring water from the bark to the xylem. Although some water taken up by the bark during the night may move into the xylem, some is also evaporated back to the atmosphere during the following early morning, as we can often see slightly increased branch transpiration during the mornings (Figures 4–5).

Notably, days with indications for water condensation in the measurement system during the previous night were excluded from the data as part of the data filtering process (see in more detail from the Material and Methods). We assumed that water condensed in the tubing in these cases and caused a high and short peak in the bark transpiration data on mornings after the atmospheric conditions allowed the tubing to dry. Eliminating nights with condensation problems reduced the number of remaining observations of water uptake, and our results probably underestimate the actual night time water uptake during the study period.

### The Effect of Irrigation Treatment on the CO_2_ Emission Through Bark

CO_2_ emission through the bark was measured to compare the diurnal and seasonal dynamics of bark transpiration and CO_2_ emission at the control and irrigated plots. This comparison is interesting, as we hypothesised the transpiration through the lenticels in the bark to be a physical process that cannot be actively controlled by the plant and is thus not affected by the plant’s water status and physiological activity, whereas we hypothesised CO_2_ emission through the bark to be strongly linked to physiological activity through respiration, bark photosynthesis and xylem transport. Unlike transpiration, CO_2_ emission through the bark was higher in the irrigated than in the control trees, both during days and nights. Daytime bark CO_2_ emission consisted of respiration, photosynthesis and CO_2_ transported in the xylem transpiration stream, whereas during dark nights, with a negligible transpiration stream, bark CO_2_ emission can be assumed to consist of respiration only. This can be seen in the results of the irrigated plot as higher stem CO_2_ emission rate during nights than during days for a given temperature, whereas there was no clear difference between nights and days in the control plot (Figure 8). This can be explained by the difference in the daily transpiration streams and potentially also the daily bark photosynthetic rates between the control and irrigated plots. The daily maximum CO_2_ emission rate typically occurred in the afternoon, later than the maximum in bark transpiration.

Branch temperature, *via* its effect on respiration, was apparently the main driver for the bark CO_2_ emission rates in this study. Previous studies have shown varying results on the response of bark respiration to irrigation depending on the severity of the drought stress (Cui et al., 2017; Maier, 2001; Salomón et al., 2019; Saveyn et al., 2007a; Stockfors and Linder, 1998). Drought stress was severe at our control plot, as seen from the shoot gas exchange data, indicating that the stomata were nearly closed from June to September. CO_2_ emission through the bark decreased strongly from June to August at the irrigated plot, whereas no change occurred in the control plot. From August to November, stem CO_2_ emission decreased at both plots. Many field studies show respiration per given temperature to be higher during the growing season than in other seasons due to growth respiration (Chan et al., 2018; Maier, 2001; Stockfors and Linder, 1998; Zha et al., 2004). The active growing season at our site ends in April, before the drier season begins (Atzmon et al., 2004; Preisler et al., 2020), and thus, the respiration component in our data set is mainly maintenance respiration. The difference between the irrigated and control plot was largest in June and July, suggesting that the irrigated trees experienced higher metabolic activity than the control trees, especially during those months. Thus, the differences seen in the CO_2_ emission through the bark were likely caused by metabolic activity rather than bark characteristics.

## Conclusion

This is the first study to report diurnal dynamics of transpiration through bark in different seasons with continuous gas exchange measurements in dry conditions. These results highlight that transpiration through bark is a passive process not affected by plant metabolic activity, unlike bark CO_2_ emission, and is not affected by the active control of lost water, unlike transpiration from needles. The role of water loss through bark is large during drought, and especially in conditions with high evaporative demand. This should not be neglected when considering the tree water balance, conductance and hydraulic aspects, such as possible hydraulic failure in *Pinus halepensis* trees, during periods when stomatal conductance is low. However, our results also demonstrated that water loss through the bark may be somewhat alleviated by nocturnal water uptake in conditions where the air becomes regularly saturated during the night.

## Data Availability Statement

The raw data supporting the conclusions of this article will be made available by the authors, without undue reservation.

## Author Contributions

AL and YP planned the study. Measurements were conducted by AL, YP, and IO. Bark data were pre-analysed by AL and shoot data by IO and YP. AL analysed the results and had the main responsibility for writing the manuscript, but all authors contributed to the writing. All authors contributed to the article and approved the submitted version.

## Funding

This work is supported by the Academy of Finland grants 310375, 337549 and 342930, and the Tyumen region government in accordance with the Program of the World-Class West Siberian Interregional Scientific and Educational Centre (National Project “Nauka”). Research at Yatir research site was partly funded by the Israel Science Foundation (ISF 1976/17), the NSFC-ISF (grant 2579/16) and the Keren Kayemet LeIsrael (KKL). The long-term operation of Yatir Forest Research Field Site is supported by the Cathy Wills and Robert Lewis Program in Environmental Sciences.

## Conflict of Interest

The authors declare that the research was conducted in the absence of any commercial or financial relationships that could be construed as a potential conflict of interest.

## Publisher’s Note

All claims expressed in this article are solely those of the authors and do not necessarily represent those of their affiliated organizations, or those of the publisher, the editors and the reviewers. Any product that may be evaluated in this article, or claim that may be made by its manufacturer, is not guaranteed or endorsed by the publisher.
