# Supplementary material for: Bark Transpiration Rates Can Reach Needle Transpiration Rates Under Dry Conditions in a Semi-arid Forest
Source: Front Plant Sci. 2021 Dec 20;12:790684. doi: 10.3389/fpls.2021.790684 (PMC8721219; doi:10.3389/fpls.2021.790684)
Supplement: Supplementary file 1 [file Data_Sheet_1.PDF]

Supplementary Material

Article: Bark transpiration rates can reach needle transpiration rates under dry conditions in a semi-arid forest

Authors: A. Lintunen, Y. Preisler, I. Oz, D. Yakir, T. Vesala and T. Hölttä

Description: This supplementary material includes data on example day dynamics of solar radiation in shoot cuvettes of different sample trees, shoot cuvette temperatures during cuvette closures, and analysis on the effect of cuvette warming due to greenhouse effect during days on the driving force for bark evaporation.

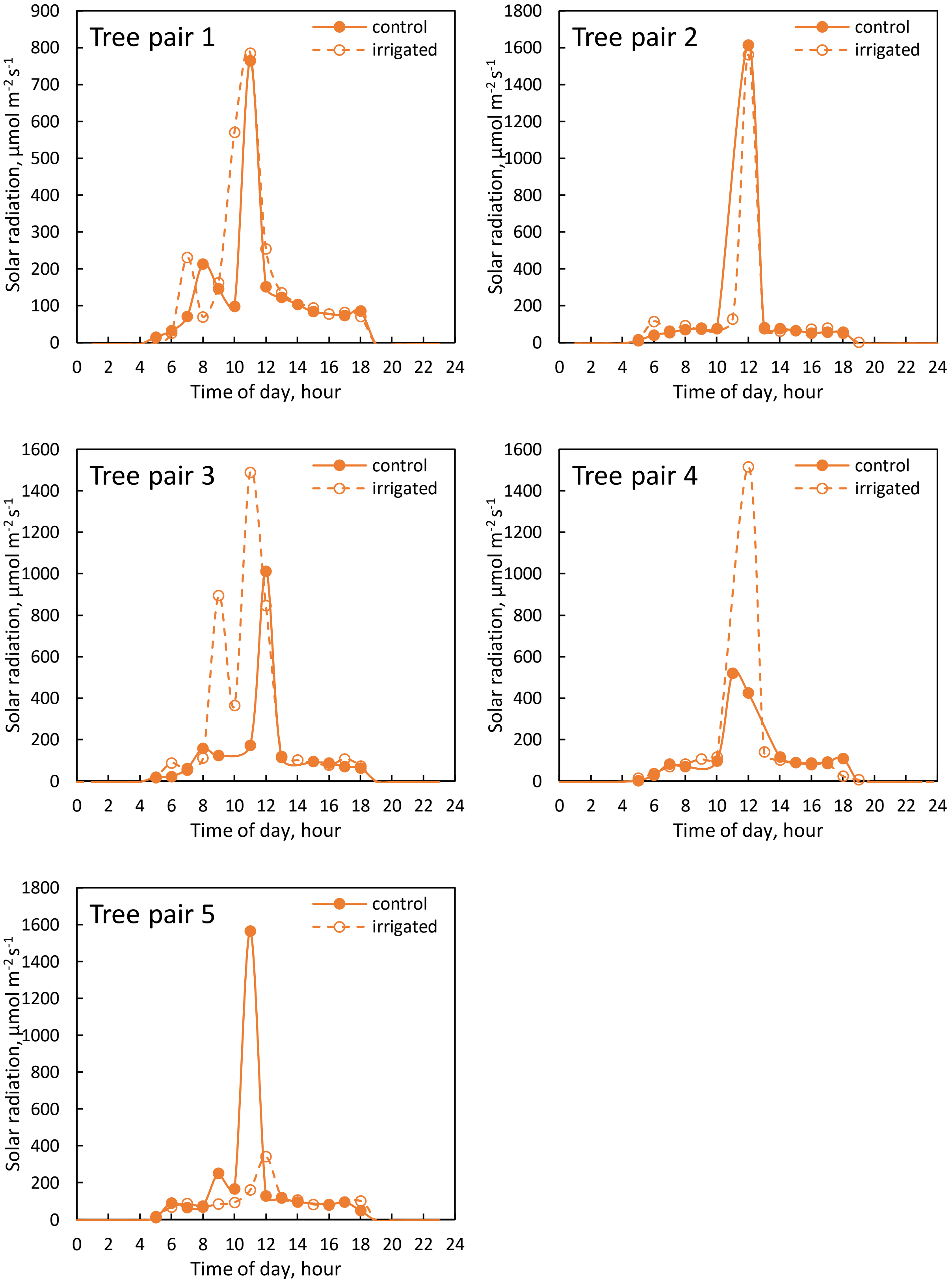

Figure S1. Solar radiation measured (PQS 1, Kipp & Zonen, Delft, The Netherlands) from the shoot cuvettes as µmol photons per m^2 per s on 24th of June in 2019.

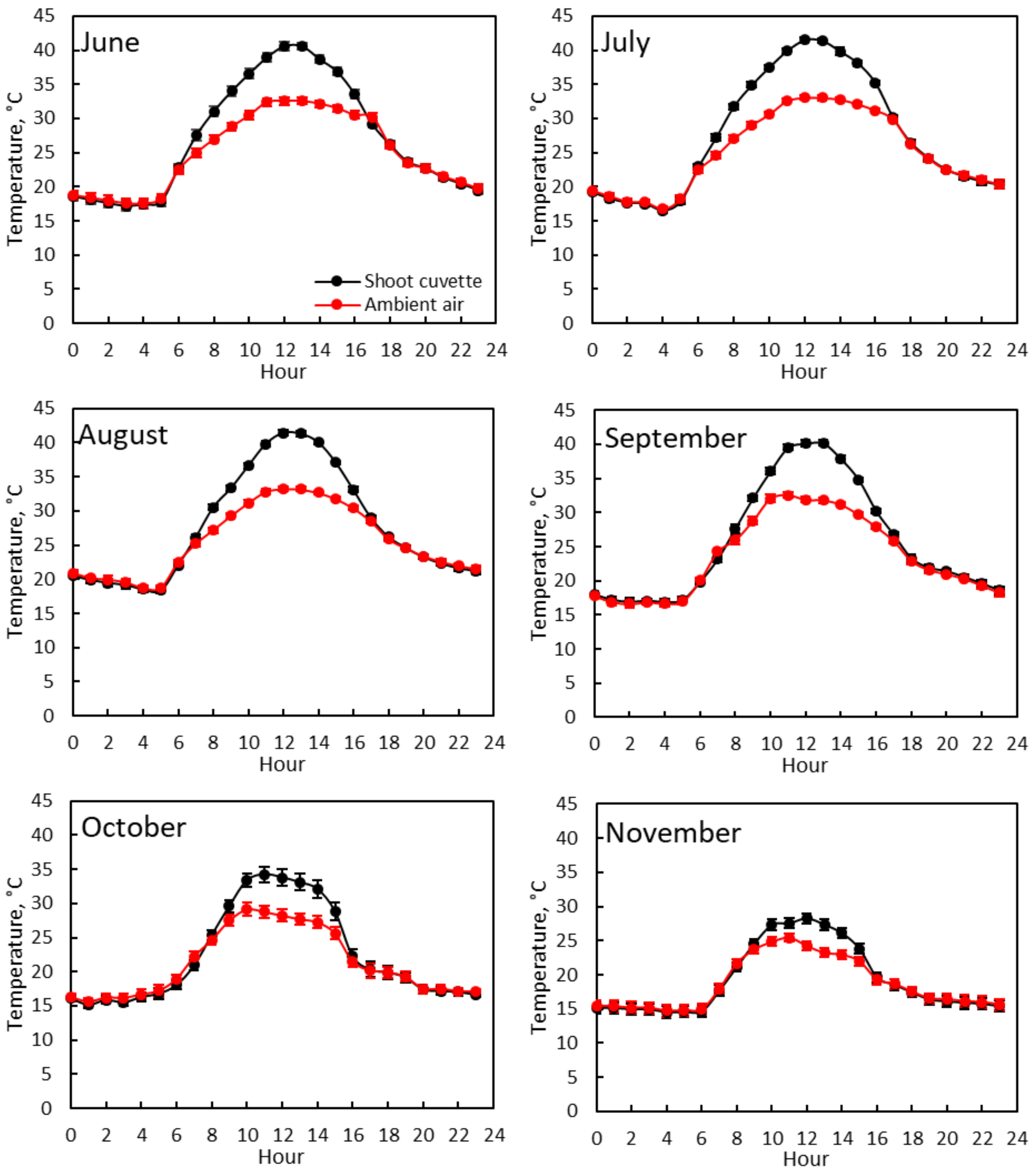

Figure S2. Temperature of ambient air (red colour) and air inside the shoot cuvettes during cuvette closure (black colour) for each month between June and November in 2019. Each subfigure represents hourly averages for one month (tree pair 5). Error bars show standard error.

Analysis on the effect of cuvette warming due to greenhouse effect on the driving force for bark evaporation:

We tested how sensitive the calculation of the driving force for water exchange through the bark is to elevated temperature of the cuvette and branch due to solar irradiation. We assumed that the branch and branch cuvette temperatures were equal to each other, and that the relation between ambient air temperature and the branch/cuvette temperatures followed the same relation as the temperature between ambient air temperature and the shoot cuvette temperature shown in Fig S3.

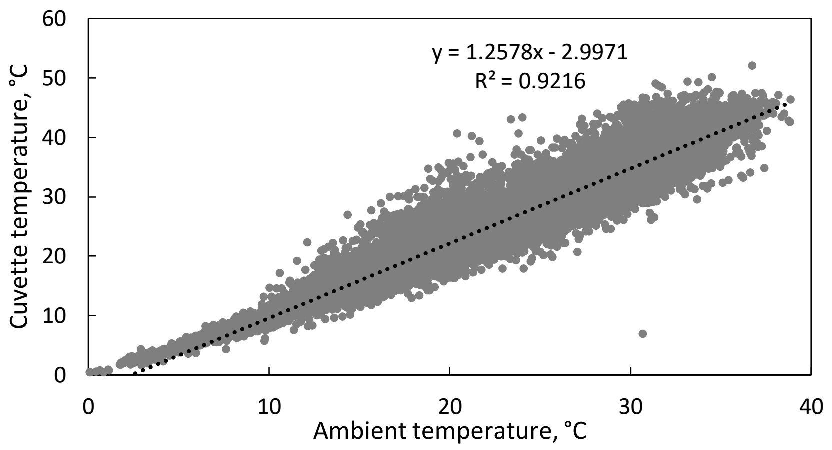

Figure S3. The measured relation between ambient air temperature and shoot cuvette temperature from June to November.

Using the regression equation shown in Fig. S3 resulted in the following relation (Fig. S4) between the driving force calculated using the assumption that branch and branch cuvette temperatures were the same as air temperature (x-axis) and the predicted driving force using the regression equation in Fig. S2 (y-axis). The sensitivity analysis demonstrates that the error in the calculation of the driving force is small, when it was assumed that the branch and the branch cuvette temperature are equal to each other, even if elevated above ambient air temperature.

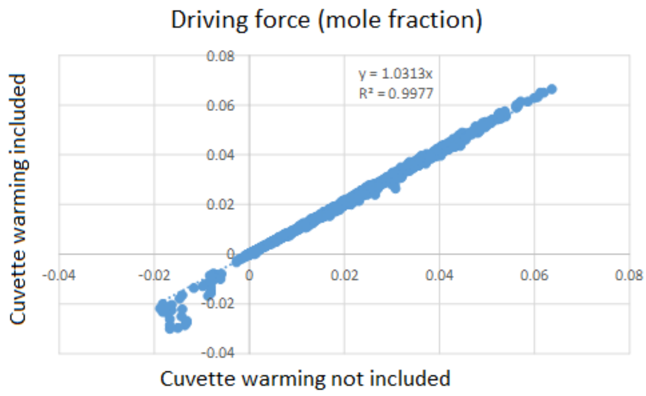

Fig S4. The predicted relation between from driving force for water exchange through the bark calculated using the assumption that both branch and branch cuvette temperature are equal to ambient air temperature (x-axis) and the assumption that both branch and branch cuvette temperatures are elevated with respect to ambient air temperature following the relation shown in Fig. S3 (y-axis).
